# Supplementation of *Boswellia serrata* and *Salix alba* Extracts during the Early Laying Phase: Effects on Serum and Albumen Proteins, Trace Elements, and Yolk Cholesterol

**DOI:** 10.3390/ani12162014

**Published:** 2022-08-09

**Authors:** Giulia Andreani, Thomas Dalmonte, Alessandro Guerrini, Caterina Lupini, Micaela Fabbri, Enea Ferlizza, Gloria Isani

**Affiliations:** 1 Department of Veterinary Medical Sciences, University of Bologna, Via Tolara di Sopra 50, Ozzano dell’Emilia, 40064 Bologna, Italy; 2 Interdepartmental Centre for Agri-Food Industrial Research (CIRI Agrifood), University of Bologna, Cesena, Piazza G. Goidanich 60, 47521 Cesena, Italy; 3 Department of Environmental Sciences and Policy, University of Milan, Via Celoria 10, 20133 Milan, Italy; 4 Department of Experimental, Diagnostic and Specialty Medicine, University of Bologna, via Belmeloro 8, 40126 Bologna, Italy

**Keywords:** protein electrophoresis, SDS–PAGE, SPE–AGE, phytoextracts, albumen protein, chicken serum proteins, iron, zinc

## Abstract

**Simple Summary:**

Consumers’ attention to sustainability and animal welfare has increased, strengthening the demand for eggs produced through alternative and extensive farming methods. At the same time, the need to reduce antibiotics has fostered the use of alternative dietary supplements. The aim of this research was to study the effects of botanical extracts from *Boswellia serrata* (*Bs*) and *Salix alba* (*Sa*) on serum and albumen proteins, serum iron and zinc, and egg cholesterol in Leghorn hens, during the critical phase of the onset of laying. The supplementation did not alter the protein profile of egg albumen and the cholesterol content of egg yolk. For the serum and albumen protein profile, no significant differences were observed between control and supplemented hens. Overall, this study confirms that the dietary supplementation with phytoextracts did not negatively affect the physiological variations in serum proteins and, therefore, can be safely used as a treatment to prevent inflammatory states during the early laying phase.

**Abstract:**

Extracts from *Boswellia serrata* (*Bs*) and *Salix alba* (*Sa*) are used as supplements in poultry feed. The aims of this research were to study the possible effects of dietary supplementation with *Bs* and *Sa* extracts on serum and albumen proteins, zinc and iron, and yolk cholesterol content in Leghorn hens during the critical phase of the onset of laying. A total of 120 pullets, 17 weeks of age, were assigned to two groups (control (C) and treated (T), n = 60 each). The T group received a supplement containing *Bs* (5%) and *Sa* (5%) for 12 weeks. The study lasted 19 weeks. Serum proteins were fractionated using agarose gel electrophoresis (AGE) and SDS–polyacrylamide gel electrophoresis (SDS–PAGE). Trace elements were determined in serum using atomic absorption spectrometry, and yolk cholesterol was determined using a colorimetric test. No significant differences were observed between control and supplemented hens for the analyzed biochemical indices. Moreover, the supplementation with phytoextracts did not negatively affect the physiological variations in serum proteins; therefore, it can be safely used as a treatment to prevent inflammatory states at onset and during the early laying phase.

## 1. Introduction

Over the last few years, consumer demand has gradually focused on product quality, which includes both the intrinsic characteristics and procedures of the productive process. Recently, consumers’ concerns about sustainability and animal welfare have increased [1,2,3], strengthening the demand for eggs and meat produced through alternative and extensive farming methods [4]. Moreover, the shift away from antibiotic use has fostered research on alternative control methods and dietary solutions aiming to improve animal health and welfare.

A wide range of feed additives, including those obtained from plants, may fall under the category of phytogenic feed additives. The European Union Register of Feed Additives [5] reported that extracts of *Boswellia serrata* and *Salix alba* can be used as additives in animal diets. The resin of *B. serrata* (*Bs*) is widely used for the treatment of inflammatory diseases, including those affecting the gastrointestinal tract, due to the bioactive compounds contained therein, particularly boswellic acid [6,7]. The supplementation with *Bs* in broiler diets improves the digestibility of nutrients due to the microbiological stabilization of the small intestine [8,9] and leads to hypolipidemic effects, with an improvement in the quality of meat [10,11]. Like *Bs*, the bark of *S. alba* (*Sa*) is used for the treatment of chronic and acute inflammation, infection, pain, and fever. The pharmacological activity is attributed to its main component, salicin, a precursor of the anti-inflammatory acetylsalicylic acid [12]. In broilers, the salicylates derived from *Sa* have been used to reduce heat stress [13]. In hens, the use of these phytoextracts might be useful during the critical phase between the last vaccination during the pullet phase, usually carried out at the age of 13–16 weeks, and the production peak (about 30 weeks of age). In this phase, hens are subjected to high stress conditions, frequently leading to intestinal dysbiosis and inflammation. Recently, Guerrini et al. (2022) [14] reported that the use of a feed supplement containing extracts of *Bs* and *Sa* is safe and can have beneficial effects on Leghorn hens during the critical pre-laying and early laying phases. Therefore, in this research, we sought to further study the effects of supplementation on blood serum and egg albumen proteins using a proteomic approach.

Gel electrophoresis is widely used to analyze protein-rich samples, from agarose gel electrophoresis used in clinical settings to separate serum proteins (SPE–AGE) to the more sensitive mono- or bidimensional sodium dodecyl sulfate–polyacrylamide gel electrophoresis (SDS–PAGE) used to separate complex protein mixtures in different biological samples [15,16,17]. Despite its routine use in domestic and farm mammals and its increasing popularity in non-conventional avian species [18], SPE–AGE has rarely been applied to poultry. The lack of standardization of fraction separation and information regarding the protein composition makes the use of this diagnostic technique in poultry limited. To the best of the authors’ knowledge, no data are present in the literature in terms of the use of this approach in laying hens. In these animals, the onset of productive phase is accompanied by major physiological changes, which determine the modifications of serum proteins in response to hormones. One of these proteins is vitellogenin, a complex phosphoglycolipoprotein produced by the liver following estrogen stimulus. Recently, Kaab et al. (2019) [19] used SDS–PAGE and reported an increase in vitellogenin and apolipoprotein-B in the sera of hens concomitant with the onset of laying. 

The aims of this study were to: (*i*) evaluate the effects of a diet supplemented with botanical extracts of *Bs* and *Sa* on serum proteins and iron and zinc concentrations during the critical phases of pre-laying and early laying in Leghorn hens; for this purpose, samples collected during the research reported by Guerrini et al. (2022) [14] were used; (*ii*) evaluate if a diet supplemented with botanical extracts of *Bs* and *Sa* modifies the profile of albumen proteins and yolk cholesterol content.

## 2. Materials and Methods

### 2.1. Animals, Study Design, and Sampling

This study is part of a Research Project approved by the Italian Ministry of Health N° 602/2021-RP and by the Ethical Committee of the University of Bologna (Protocol N° 1139). In total, 120 pure-bred white Leghorn pullets (17 weeks of age/120 days old) were randomly assigned to 2 experimental groups, a control group (C) and a treated group (T), with 60 pullets each. Pullets were certified *Salmonella*-free, vaccinated for Newcastle Disease and Infectious Bronchitis, and non-beak-trimmed. The animals were raised on a free-range farm located in Mugello (43°59′20.2″ N 11°27′58.1″ E, Florence, Italy) for 19 weeks of trial, from mid-August to December 2021. Supplementation with *Bs* and *Sa* was administered to the T group during the first 12 weeks of the trial. Four blood samplings were performed: at the start of the trial (t0), after 5 weeks of supplementation (t1), at the end of supplementation (t2, 12 weeks), and at the end of the trial (t3, 19 weeks). The hens of the T group started laying eggs at the 7th week of supplementation, and the C group started 6 days later. Blood samples (2.5 mL) were collected from the brachial vein of 8 identified hens, using a sterile syringe with a 23G-0.60 mm needle, in clean centrifuge tubes. At each experimental time point, the same hens were sampled. The serum was obtained after centrifugation at 1500× *g* at 4 °C for 10 min and stored in 2 mL plastic vials at −80 °C until analysis. 

During the 10 weeks of laying, 5 eggs were collected each week from the T and C groups, separated in albumen and yolk, which were separately pooled and stored at −20 °C for protein analysis and cholesterol quantification, respectively.

### 2.2. Management and Diet 

The two experimental groups were managed following Guerrini et al. (2022) [14], where detailed information on animal management and performance can be found. The animals were fed with a commercial diet (*Cargill* s.r.l. feed, Table 1) that was offered *ad libitum*, supplemented for 12 weeks for the T group, with 300 g of supplement/100 kg feed (0.3%). The supplement contained 5% of dry extract of *B. serrata* (*Bs*) and 5% of dry extract of *S. alba* (*Sa*). Water was provided *ad libitum*. The C group received the commercial diet without supplement throughout the trial, while from the 13th to 19th week (the end of the trial), the T group received the commercial diet without supplement. Animals were not subjected to medical treatment during the trial to eliminate interference with the normal feed intake. The outside paddock consisted of an activity area without pasture or vegetation.

### 2.3. Roche Scale for Yolk Color Evaluation

The Roche scale (Rs) Yolk Color Fan comprises a range of yolk colors from 1 to 15 color points. Five eggs/group were collected every week, for evaluation of the Roche scale. The yolk color score was individually attributed to each single egg, in natural laboratory light conditions.

### 2.4. Serum Protein Separation Using Agarose Gel Electrophoresis (SPE–AGE)

Serum samples (10 µL) were fractionated on 0.8% agarose gel (Hydragel, Sébia, Lisses, France). The electrophoresis was run on an automated system (Hydrasis, Sébia, Lisses, France) following the manufacturer’s instructions. Gels were stained using amido-black. Stained gels were digitalized with a scanner yielding the densitometric profile (Phoresis 6.1.2 software, Sébia, Lisses, France) and the relative percentage of each protein fraction; then, the densitometer calculated the absolute value of the different protein fractions by multiplying the total protein concentration by the corresponding fractional percentage. For fraction identification, in the absence of reference data for hens, the profile of broiler serum proteins was used as a reference. Total protein concentration was determined using a commercial kit (Olympus Systems Reagents, Brea, CA, USA), with an automated biochemical analyzer (Olympus AU400, Mishima Olympus Co. Ltd., Shizuoka, Japan).

### 2.5. Serum and Albumen Protein Separation Using Sodium Dodecyl Sulfate Polyacrylamide Gel Electrophoresis (SDS–PAGE)

Samples (15 µg of serum protein and 10 µL of albumen previously diluted 1:100 (*v*:*v*) with bidistilled water) were loaded onto 4–12% polyacrylamide pre-cast gels (NuPage/Thermo Fisher Scientific, Waltham, MA, USA). Electrophoresis was carried out in a Novex Mini-Cell (Invitrogen) 4-(3-sulfonatopropyl) morpholin-4-ium buffer (MOPS; NuPage/Thermo Fisher Scientific, Waltham, MA, USA), containing lithium dodecyl sulfate (LDS). Each gel was also loaded with standard proteins of known molecular weight (SeeBlue Pre-Stained Standard). The electrophoresis system was connected to a power supply (Power Pack Basic—Bio-Rad, Hercules, CA, USA) at a constant voltage of 200 V. The gels were stained with Quick Coomassie Stain (Protein Ark, Sheffield, UK). After destaining, each gel was digitalized using ChemiDocMP (Bio-Rad, Hercules, CA, USA), and the pherograms were obtained using the ImageLab 5.2.1 software (Bio-Rad, Hercules, CA, USA). The ImageLab software estimates the volume of protein bands based on pixel density. In serum samples, the quantification of the protein bands was performed, knowing the content of total proteins (15 µg) loaded on the gel for each sample and the percentage of the band determined after densitometric analysis, a process applied in clinical routine with agarose gel electrophoresis.

### 2.6. Iron and Zinc Determination in Sera 

To avoid contamination, all the reagents were carefully handled, and polyethylene disposables were thoroughly washed with HCl 1 N under a fume hood. All the reagents were from Merck (Darmstadt, Germany); the acids were of Suprapur grade. Samples of sera were diluted 1:10 with bidistilled water and were directly analyzed using a flame atomic spectrophotometer (AAnalyst 100, PerkinElmer, Waltham, MA, USA). The accuracy of the method was evaluated by analyzing an international standard (ERM^®^-BB422 fish muscle). The concentrations found with the method used in this study fell into the certified uncertainty interval given by the ERM, corresponding to a 95% confidence level. The detection limits were 0.04 μg mL^−1^ and 0.01 μg mL^−1^ for iron and zinc, respectively. Element concentrations were reported as μg mL^−1^.

### 2.7. Cholesterol Quantification in Egg Yolks

A commercial test kit (Cholesterol Quantification Assay Kit, Sigma-Aldrich^®^; Darmstadt, Germany) was used for the determination of total cholesterol in egg yolk. During the 10 weeks of laying, 5 eggs were collected from C and T groups. Yolks were separated from albumens, pooled, and stored at −20 °C. Following Pasin et al. (1998) [20], 3 g of the liquid yolk were solubilized in 27 mL of 5% NaCl solution to obtain a 10-dilution factor. Afterwards, samples were homogenized with UltraTurax and gently stirred for 2 h. Subsequently, 100 μL of each sample were diluted with 900 μL of 5% NaCl solution to obtain a 1:100 final dilution factor and used as a working sample. Cholesterol was determined following the manufacturer’s instructions, in a microplate reader (Tecan Trading, Männedorf, Switzerland).

### 2.8. Statistical Analysis

Shapiro–Wilk and Lilliefors statistical tests were used to determine the distribution of data. To assess the homoscedasticity, Levene and Brown–Forsythe tests were performed. The Friedman test was used to determine significant differences among time points within the same group for serum and albumen SDS–PAGE, serum AGE, and cholesterol quantification in yolk samples. The Nemenyi post hoc test was then performed to evaluate the significant difference when the Friedman test was significant. The comparison between the C and T groups at the same time point was performed using the Mann–Whitney test. All tests were conducted using a statistical software program (RStudio-1.2.1335 Statistical and R, R version 3.4), and the results were considered statistically significant at *p* < 0.05. Data are reported as mean ± standard deviation (SD). 

## 3. Results and Discussion

### 3.1. Roche Scale

Compared with the 1/15 scale, the eggs of the T group recorded an average score of 10.36 ± 0.32, while the eggs of the C group recorded a score of 10.5 ± 0.58 color scale points. No statistically significant differences were found. 

### 3.2. SPE–AGE 

**Figure 1:**
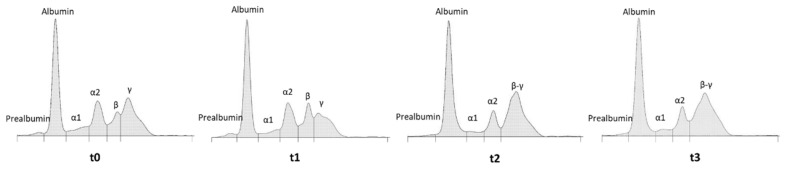
Representative electropherograms of serum proteins using agarose gel electrophoresis (AGE) obtained from the same control hen at different experimental time points: t0, start of the trial; t1 (5th week), before the start of laying; t2 (12th week), end of the supplementation; and t3 (19th week), end of the trial.

In non-mammalian species, serum protein electrophoresis on agarose gel might be considered a useful clinical tool with a still underexploited diagnostic and prognostic potential. However, there are no defined criteria for the identification of different protein fractions in many species, including *Gallus gallus*. Recently, a study conducted by Tóthová et al. (2019) [21] reported the separation of agarose gel in broiler serum proteins using the same protocol and platform as that used in the present research. Therefore, three serum samples obtained from healthy broilers were analyzed before hen samples and pherograms were compared with those obtained by Tóthová et al. (2019) [21]. Similar pherograms were obtained, and protein fractions were separated accordingly. At t0, based on the profile obtained for broilers, five major fractions were also separated in hens, including albumin, the predominant, most anodic band, followed by α, β, and γ globulins. A faint band of prealbumin was also present before the albumin band in all the examined samples. The alpha zone was clearly divided into α1 and α2, while it was not possible to separate β1 and β2 subfractions (Figure 1, t0).

It is well-recognized that the AGE applied to serum and plasma samples suffers from differences due to commercial platforms and reagents [22]. However, despite subtle differences in electrophoretic profiles, Cray (2021) [18], using a different platform (Helena SPIFE 3000 system), reported the same fractions (prealbumin, albumin, α1, α2, β, and γ globulins) in the electropherograms of chicken sera that were found in this study. Therefore, taken together, these data can be considered a first attempt to define a standard profile for serum proteins in healthy chickens.

In the control hens, qualitative and quantitative variations were evident over time, from t0 to t3, due to the onset of the laying phase, which is accompanied by physiological changes and variations in serum proteins. As an example, the pherograms obtained from the same specimen at the different experimental time points are reported in Figure 1. The complete data and statistical analysis are reported in Table 2. Between t1 and t2, the hens started the laying phase, and a significant (*p* < 0.05) increase in albumin concentration from 15.0 ± 1.6 (t0) to 21.9 ± 0.5 g L^−1^ (t3) was measured in the control group. An increasing trend of serum albumin was also reported by Gyenis et al. (2006) [23] for Hy-Line W98 and Hy-Line Brown hens from the 6th to 31st week, during the critical phases including pre-laying, the onset of laying, and the phase reaching the production peak. In the control group, a significant decrease (*p* < 0.05) was detected for α2 globulins from 7.1 ± 0.9 to 5.2 ± 0.05 g L^−1^. However, the most marked differences were present in β and γ globulins, with a fusion of these two zones (Figure 1, t2 and t3), likely due to an increase in the β zone. Therefore, starting from t2, it was not possible to separate the peaks of β and γ globulins. Hypotheses for these variations are discussed below.

Supplemented hens presented the same variations over time. No significant differences were detected between the two experimental groups, with the sole exception of albumin at t1. At this time point, the control hens had a concentration of serum albumin significantly higher than the hens supplemented with the phytoextracts. 

### 3.3. SDS–PAGE of Serum Proteins

**Figure 2:**
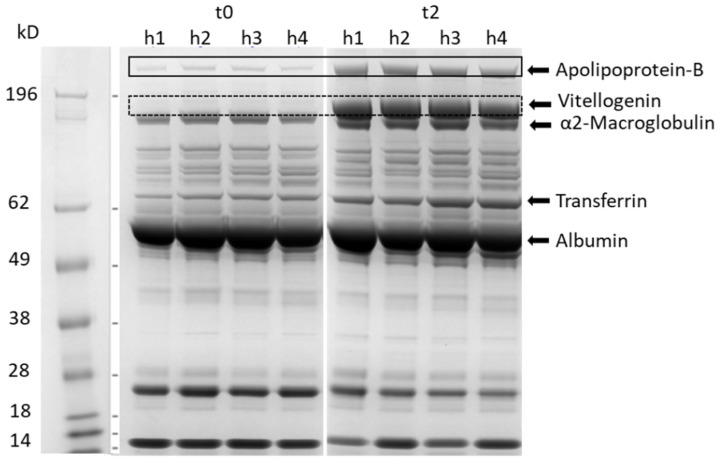
Representative gel of serum proteins using SDS–PAGE obtained from the same 4 control hens (h1–h4) at t0, start of the trial (before the laying phase) and at t2, 12 weeks later (hens in laying phase). The molecular mass (kD) of marker proteins is shown to the left of lane 1. Putative proteins are identified based on the MM and the position on the gel.

Information on proteins present in the different globulin fractions is fragmentary in chickens, but we can hypothesize that they are the same as those in mammals due to their essential and shared physiological and biochemical role. To shed more light on these proteins and their possible changes during the laying phase, serum samples were further fractionated using SDS–PAGE. The representative gels of serum samples obtained from the control hens at t0 (before laying) and at t2 (hens in the laying phase) are reported in Figure 2. The band profiles obtained in Leghorn hens are the same as those reported by Kaab et al. (2019) [19], who identified 11 abundant proteins via mass spectrometry in the sera of Lohmann Brown hens after SDS–PAGE fractionation. Therefore, the identity of some of the bands obtained in this research can be hypothesized based on the apparent molecular mass (MM) and comparing the data with those reported by Kaab et al. (2019) [19]. At t0, protein profiles revealed a common pattern in all the analyzed samples, characterized by the presence of a high-abundance band at an apparent MM of 55 kD; this band can be identified as putative albumin, in accordance with the data obtained using agarose gel electrophoresis (Figure 1). Over time, from t0 to t3, qualitative and quantitative differences were recorded, mirroring the onset of the laying phase. 

**Figure 3:**
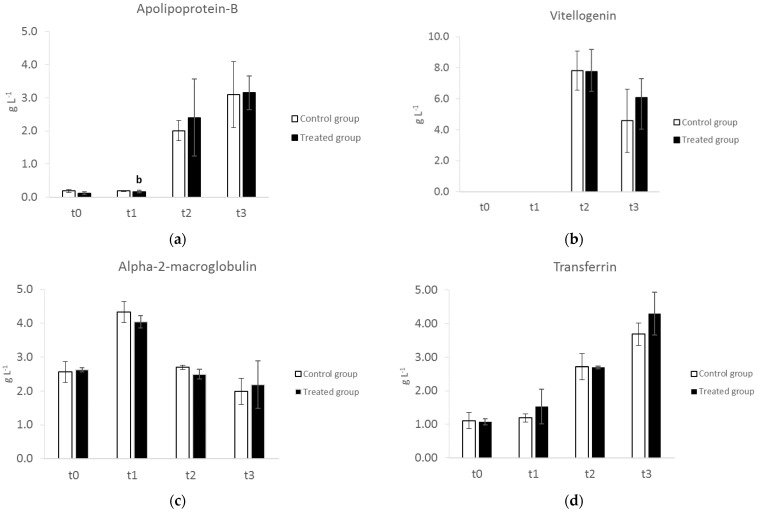
Variation in selected serum proteins in control and supplemented hens at different time points: t0, start of the trial; t1 (5th week), before the start of laying; t2 (12th week), end of the supplementation; and t3 (19th week), end of the trial. The concentrations were obtained after densitometric analysis of protein bands and are expressed as g L^−1^ (n = 4). Bars sharing the same symbol (control hens) or the same letter (treated hens) are significantly different (*p* < 0.05).

Besides albumin, at t0, one other high-abundance protein band had an apparent MM of 160 kD and can be identified as α2 macroglobulin (α2M) (Figure 2 and Figure 3c). Vertebrate α2Ms are large tetrameric glycoproteins consisting of four identical subunits, the main function of which is to inhibit the circulating proteases resulting in different biological properties, such as antibacterial and anti-inflammatory activity [24]. In chickens, this protein, previously known as ovostatin, is present also in the egg albumen [25]. The intensity of this band significantly decreased (*p* < 0.05) over time from t1 to t3 (Figure 3c). Similarly, Kaab et al. (2019) [19] reported a decrease in α2M, starting from the onset of the laying period, suggesting an increased demand for this protein to be incorporated into the eggs. The decrease in the α2 zone observed using AGE might be in part due to the decrease in α2M, which is one of the main components of the α2 zone, together with haptoglobin.

Starting from t2, in parallel with the onset of the laying phase, the upper part of the gel showed two additional high MM proteins at >200 and 175 kD, respectively (Figure 2). These two proteins can be identified as apolipoprotein-B (Apo-B) and vitellogenin (Vg), likely Vg2, which is the most abundant Vg isoform. Accordingly, Kaab et al. (2019) [19] reported in Lohmann Brown layers that these two proteins significantly increased by the 17th week and reached a peak by the 23rd week just before the production peak. Apo-B and Vg are essential for egg formation, contributing to the lipid and protein components of eggs. Apo-B is the main protein of LDLs and VLDLs and, in mammals pherograms, appears in the beta zone on agarose gels. In laying hens, VLDLs are devoted to the transport of triacylglycerols from the liver to the oocytes. With the onset of egg production, lipoprotein production in the liver is shifted from generic VLDL to VLDLy (yolk-targeted) because triacylglycerols, which are accumulated and stored in egg yolk, are needed to satisfy the embryo’s energy requirement for hatching [26]. Vitellogenins are the most relevant source of nutrients for the developing embryo in non-mammalian vertebrates. These proteins are produced by the liver after estradiol stimulus, secreted into the bloodstream, and taken up in the oviduct by receptor-mediated uptake [27,28]. The location of Vg in the globulin zone of agarose gel pherograms is still unknown. However, it can be hypothesized that the fusion of beta and gamma zones at t2 and t3 might be in part due to the presence of Vg. The presence of vitellogenins in the egg-laying females of non-mammalian vertebrates should be considered when analyzing pherograms in clinical settings.

Based on the position on the gel and the MM, the band at 72 kD can be identified as transferrin. Transferrins are iron-binding glycoproteins that bind and transport ferric ions in biological fluids. Due to this binding specificity, transferrins have antibacterial activity, making iron unavailable for bacterial growth [29]; at the same time, in laying hens, they are essential for iron delivery to the eggs. Watanabe and Iwasaki (1985) reported an increase in transferrin concentrations in the sera of laying hens, suggesting an important function associated with iron metabolism in egg laying [30]. Accordingly, a significant increase (*p* < 0.05) in this protein was found from t0 to t3 (Figure 2 and Figure 3d) in this study.

The supplemented hens presented the same profile and the same variations in serum proteins over time (Figure 3) as the control hens. No significant differences in serum proteins were detected between the control and supplemented hens, indicating that the supplementation with phytoextracts did not negatively affect the serum protein profiles.

### 3.4. Iron and Zinc Concentrations in Serum

**Figure 4:**
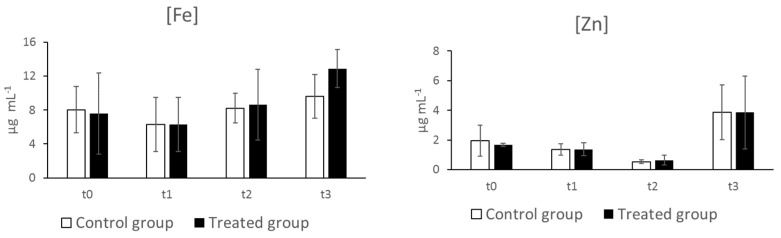
Iron (Fe) and zinc (Zn) concentrations in sera of control (C) and supplemented hens (T). Data are reported as mean ± SD (n = 8) and expressed as μg mL^−1^.

In hens, the trace element dynamics in serum during the laying phase is still an underexplored field, and the reference intervals are also not defined. Therefore, iron and zinc concentrations were analyzed at t0 and at different time points to evaluate the possible variations related to egg laying and supplementation. Iron concentrations determined in this study in the control hens varied from 8.03 ± 2.75 (t0) to 9.59 ± 2.57 (t3) µg mL^−1^ (Figure 4) in accordance with the increase of transferrin. These concentrations are similar to those reported in turkey laying hens by Richards (1989) [31], while they are higher than the values reported by Sarlak et al. (2021) [32] in Shaver White laying hens. 

The zinc concentrations determined in this study in the control hens varied from 1.95 ± 1.05 (t0) to 3.86 ± 1.85 (t3) µg mL^−1^. In broilers, similar concentrations (1.68–2.07 µg mL^−1^) (1.98 µg mL^−1^) were reported by Bartlett and Smith (2003) [33] and by Olukosi et al. (2018) [34], respectively, while higher concentrations (10.53 ± 0.26 µg mL^−1^) were reported by Sohail et al. (2011) [35]. 

No significant variations were detected among different time points, despite an increasing trend from t1 to t3 for iron and from t2 to t3 for zinc, probably associated with the onset of egg laying between t1 and t2. In fact, egg formation requires the mobilization of the stored iron from the liver and its transport to the eggs, which should contain an adequate amount of this essential element to ensure embryonic growth and development.

Iron and zinc concentrations in sera did not significantly differ between the control and supplemented hens.

### 3.5. SDS–PAGE of Albumen Proteins

**Figure 5:**
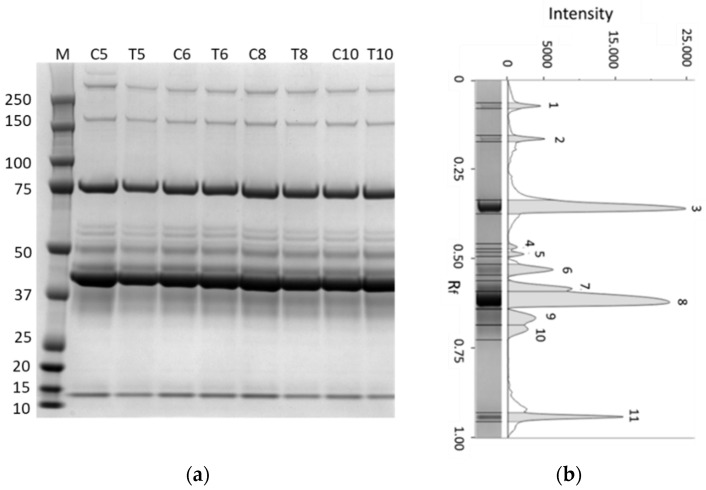
Representative gel and pherogram of egg albumen proteins separated using SDS–PAGE 4–12%: (**a**) lane 1: molecular mass marker (kD); lanes 2, 4, 6, and 8: albumens from control hens at 5th, 6th, 8th and 10th week (C5–C10); lanes 3, 5, 7, and 9: albumens from supplemented hens at 5th, 6th, 8th, and 10th week (T5–T10); (**b**) the pherogram obtained from lane 3 is reported as an example (right).

Egg albumen provides important nutrients for embryo development and contains proteins of high nutritional value. Figure 5 reports representative gel and pherograms of egg albumen proteins obtained from the control hens at different laying weeks. SDS–PAGE separation revealed 11 protein bands, ranging in the MM from >250 to 14 kD. The identity of these proteins can be hypothesized based on the apparent MM, the position on the pherogram, and comparing the profiles with those reported in the literature. 

In fact, during the last two decades, studies reported a common protein profile after SDS–PAGE for the proteome of egg albumen in hens, indicating that the most abundant proteins are ovalbumin (45 kD MM), ovotransferrin (75 kD MM), ovomucoid (28 kD MM), lysozyme (14 kD MM), and ovomucin [36,37]. The profiles obtained in the samples analyzed in this study were characterized by two abundant protein bands at 43 (band 8) and 75 kD (band 3) that can be identified as ovalbumin and ovotransferrin. These data confirmed the findings of previous studies [25,38,39], with interesting differences regarding the abundance of ovalbumin and ovotransferrin. In all the analyzed samples, and in the optimized experimental conditions in this study, a less intense band of ovalbumin associated with an evident band of ovotransferrin was detected. Wang et al. (2012) [40] reported that the abundance of ovalbumin was significantly different (*p* < 0.05) among six egg varieties and ovalbumin spots after 2DE in brown-shelled eggs were 81% higher than those of white-shelled eggs. It could be hypothesized that white-shelled eggs, such as eggs from Leghorn hens, have a lower content of ovalbumin. If confirmed by future studies, the abundance of ovotransferrin in the albumen of Leghorn hen eggs could represent an interesting source of bioavailable iron for human nutrition.

A diffuse zone containing two bands was present between 38 (band 9) and 33 (band 10) kD. These bands could be attributed to ovomucoid and ovoflavoprotein, two highly glycosylated proteins [36]. The high glycosylation degree, estimated at 25% for ovomucoid [41], could be responsible for the low migration rate in SDS–electrophoresis and the difference between the theoretical (28 kD) and measured (35 kD) MM for ovomucoid [42]. Finally, the band at 14 kD (band 11) could be identified as lysozyme, a well-known antimicrobial protein of albumen. 

Similar profiles of albumen proteins were detected in the control and supplemented hens (Figure 5).

### 3.6. Cholesterol Content in Egg Yolk

Cholesterol was measured in egg yolk at the 1st, 5th, 8th, and 10th laying week, corresponding to the start of laying, the end of supplementation (12th week of trial), 21 days from the end of supplementation (15th week of trial), and the end of the trial (19th week of trial), respectively (Table 2). The content of cholesterol detected in the eggs of the control hens, with a mean value over the 10 weeks of 12.8 mg g^−1^ of wet yolk, is in accordance with the mean value of 12.8 mg g^−1^ of yolk reported by Abou-Elkhair et al. (2018) [43] in Lohmann Brown Lite hens. A different value for cholesterol content in the eggs of Leghorn hens was reported by Panda et al. (2003) [44], with a mean value of 13.5 ± 0.15 mg g^−1^ of yolk. It is well-known that cholesterol content in yolk may be influenced by many variables, including breed, age, and housing system [45]; therefore, these differences were expected. 

Regarding the effects of feed supplementation with *Bs* and *Sa* extracts, a lower, though not significant, value for yolk cholesterol content was measured in the T group (Table 3). It is well-recognized that the extracts of *Bs* have hypolipemic effects in rats [46], humans [47], and broilers [48], but there is little information regarding their possible effects on the content of egg yolk. Abdelli et al. (2021) [49] and Panda et al. (2003) [44] reported that phytogenic feed additives and probiotic supplementation reduced the yolk’s cholesterol content. Further insights are needed to disclose the effects of *Bs* and *Sa* supplementation on the cholesterol content of eggs.

## 4. Conclusions

No significant differences in serum proteins were detected between the control and supplemented hens, indicating that the supplementation with botanical extracts of *Bs* and *Sa* did not negatively affect these biomarkers during the pre-laying and early laying phases and, therefore, can be safely used as a treatment to prevent inflammatory states during these critical phases. No significant differences in egg albumen proteins and cholesterol content in yolk were observed between the two groups, indicating that phytoextracts did not influence the quality of eggs in Leghorn laying hens. A possible effect on the content of cholesterol in eggs needs further studies and could be interesting for future applications if the lower cholesterol content is confirmed in the yolk of hens fed a diet enriched with these phytoextracts.

## Figures and Tables

**Table 1:** Feed formulation of the commercial diet (Cargill s.r.l) based on the indications of the commercial tag and composition of the complementary feed. The table was obtained from Guerrini et al. (2022) [14].

**Composition**	**Values of Nutrients** **(%/kg of Finisher Diet)**	**Additives** **(mg/kg; IU; OTU/kg)**	**Complementary Feed** **Composition (%)**
Corn; Corn gluten flour; Soybeans meal (* CP 43%); Calcium carbonate; Roasted soybeans; Rice husk; Corn gluten; Wheat bran; Soybean oil.	CP, 17%; ** CF, 5%; *** CF, 3.51%; ^!^ Cash, 13.27%; ^ǂ^ Ca, 4.02%; ^ǂǂ^ P, 0.58%; ^≠^ NaCl, 0.15%; ^+^ Ly, 0.85%; ^$^ Met: 0.33%.	Vitamin A, 9950 IU; Vitamin D_3_, 2701 IU; Vitamin E, 38 mg; Vitamin K3, 2 mg; Vitamin B1, 1.5 mg; Vitamin B2, 4.5 mg; Vitamin B6, 2.5 mg; Vitamin B12, 0.008 mg; Niacin, 35 mg; Ca-D-pantothenate, 10 mg; Folic acid, 1 mg; Biotin, 0.1 mg; Betaine hydrochloride, 250 mg; Cu, 5 mg; Anhydrous calcium iodate, 0.50 mg; Mn, 50 mg; Se, 0.075 mg; Zn, 40 mg; Cantaxantine, 2 mg; Promoters of digestion 6-phytase, 213 OTU; DL-methionine, 627 mg.	Calcium carbonate, 74.5%; Colloidal silica, 15%; *Salix alba* ^&^ DE, 5%; *Boswellia serrata* ^&^ DE, 5%; Sodium chloride, 0.35%; Magnesium carbonate, 0.15%.

* CP: crude protein; ** CF: crude fats; *** CF: crude fiber; ^!^ Cash: crude ash; ^ǂ^ Ca: calcium; ^ǂǂ^ P: phosphorus; ^≠^ NaCl: sodium chloride; ^+^ Ly: lysine; ^$^ Met: methionine; ^&^ DE: dry extract.

**Table 2:** Concentrations of serum protein fractions determined after agarose gel electrophoresis in control (C) and supplemented (T) hens at different experimental time points: t0, start of the trial; t1 (5th week), before the start of laying; t2 (12th week), end of the supplementation; t3 (19th week), end of the trial. Data are reported as mean ± SD (n = 8 for each group).

Proteins	C				T			
	t0	t1	t2	t3	t0	t1	t2	t3
Total proteins (g L^−1^)	42.7 ± 3.13 ^(1,2)^	48.4 ± 3.62 ^(1)^	48.7 ± 4.85 ^(2)^	45.9 ± 3.40	39.3 ± 2.92 ^(1,2,3)^	46.2 ± 4.11 ^(1)^	46.8 ± 4.30 ^(2)^	48.3 ± 4.10 ^(3)^
Prealbumin (g L^−1^)	0.70 ± 0.20	0.80 ± 0.40	0.70 ± 0.20	0.80 ± 0.20	0.80 ± 0.20	0.80 ± 0.10	0.60 ± 0.20	0.80 ± 0.30
Albumin (g L^−1^)	15.0 ± 1.6 ^(1,2)^	19.5 ± 1.36 ^§^	22.2 ± 1.40 ^(1)^	21.9 ± 0.50 ^(2)^	14.2 ± 3.00 ^(1,2)^	14.9 ± 1.69 ^(3,4),§^	20.9 ± 1.70 ^(1,3)^	23.5 ± 1.10 ^(2,4)^
α1 Globulins (g L^−1^)	2.30 ± 0.30 ^(1)^	2.50 ± 0.70 ^(2,3)^	1.60 ± 0.30 ^(1,2)^	1.90 ± 0.30 ^(3)^	2.50 ± 0.70 ^(1,2)^	2.10 ± 0.20	1.80 ± 0.30 ^(1)^	1.50 ± 0.30 ^(2)^
α2 Globulins (g L^−1^)	7.10 ± 0.90 ^(1,2)^	8.40 ± 1.40 ^(3,4)^	5.00 ± 0.20 ^(1,3)^	5.20 ± 0.50 ^(2,4)^	6.40 ± 0.80 ^(1)^	7.80 ± 0.50 ^(2,3)^	4.80 ± 0.09 ^(1,2)^	5.30 ± 0.40 ^(3)^
β Globulins (g L^−1^)	4.80 ± 1.5	6.60 ± 1.60	-	-	3.40 ± 1.20	7.80 ± 1.10	-	-
γ Globulins (g L^−1^)	12.3 ± 2.2	11.5 ± 2.10	-	-	11.5 ± 2.10	12.9 ± 1.90	-	-
β-γ Globulins (g L^−1^)	-	-	18.1 ± 4.30	16.2 ± 3.50	-	-	18.6 ± 3.50	17.2 ± 3.70

^1–4^ In the same row and for each group, the same superscript number indicates a significant difference among time points. ^§^ In the same row and for the same time point, this indicates significant difference between control and supplemented hens.

**Table 3:** Cholesterol content in yolk. Data are reported as mean ± SD (n = 3) and expressed as mg g^−1^.

Laying Week	C (Control Hens)	T (Supplemented Hens)
1st	12.8 ± 1.49	10.8 ± 0.81
5th	11.9 ± 1.50	11.0 ± 1.30
8th	13.4 ± 2.12	10.8 ± 1.05
10th	13.2 ± 3.12	10.1 ± 1.38

## Data Availability

The data presented in this study are available on request from the corresponding author.
